# Swine Influenza A Outbreak, Fort Dix, New Jersey, 1976

**DOI:** 10.3201/eid1201.050965

**Published:** 2006-01

**Authors:** Joel C. Gaydos, Franklin H. Top, Richard A. Hodder, Philip K. Russell

**Affiliations:** * Walter Reed Army Institute of Research, Silver Spring, Maryland, USA;; † MedImmune, Incorporated, Gaithersburg, Maryland, USA;; ‡ Northeast Center for Special Care, Lake Katrine, New York, USA;; § Albert B. Sabin Vaccine Institute, New Canaan, Connecticut, USA

**Keywords:** Influenza, military, respiratory disease, swine, perspective

## Abstract

In early 1976, the novel A/New Jersey/76 (Hsw1N1) influenza virus caused severe respiratory illness in 13 soldiers with 1 death at Fort Dix, New Jersey. Since A/New Jersey was similar to the 1918–1919 pandemic virus, rapid outbreak assessment and enhanced surveillance were initiated. A/New Jersey virus was detected only from January 19 to February 9 and did not spread beyond Fort Dix. A/Victoria/75 (H3N2) spread simultaneously, also caused illness, and persisted until March. Up to 230 soldiers were infected with the A/New Jersey virus. Rapid recognition of A/New Jersey, swift outbreak assessment, and enhanced surveillance resulted from excellent collaboration between Fort Dix, New Jersey Department of Health, Walter Reed Army Institute of Research, and Center for Disease Control personnel. Despite efforts to define the events at Fort Dix, many questions remain unanswered, including the following: Where did A/New Jersey come from? Why did transmission stop?

## Abstract 2

Published literature and events surrounding the outbreak are reviewed.

Revisiting events surrounding the 1976 swine influenza A (H1N1) outbreak may assist those planning for the rapid identification and characterization of threatening contemporary viruses, like avian influenza A (H5N1) (*1*). The severity of the 1918 influenza A (H1N1) pandemic and evidence for a cycle of pandemics aroused concern that the 1918 disaster could recur (*2**,**3*). Following the 1918 pandemic, H1N1 strains circulated until the "Asian" influenza A (H2N2) pandemic in 1957 (*3*). When in early 1976, cases of influenza in soldiers, mostly recruits, at Fort Dix, New Jersey, were associated with isolation of influenza A (H1N1) serotypes (which in 1976 were labeled Hsw1N1), an intense investigation followed (*4*).

Of 19,000 people at Fort Dix in January 1976, ≈32% were recruits (basic trainees) (*4*). Recruits reported to Fort Dix for 7 weeks of initial training through the basic training reception center, where they lived and were processed into the Army during an intense 3 days of examinations, administrative procedures, and indoctrination. At the reception center, training unit cohorts were formed. Recruits were grouped into 50-member units (platoons) and organized into companies of 4 platoons each. Units formed by week's end moved from the reception center to the basic training quarters. To prevent respiratory illnesses, recruits were isolated in their company areas for 2 weeks and restricted to the military post for 4 weeks (*4*). Platoon members had close contact with other platoon members, less contact with other platoons in their company, and even less contact with other companies.

On arrival, recruits received the 1975–1976 influenza vaccine (A/Port Chalmers/1/73 [H3N2], A/Scotland/840/74 [H3N2], and B/Hong Kong/15/72) (*4*). Other soldiers reported directly to advanced training programs of 4 to 12 weeks at Fort Dix immediately after basic training at Fort Dix or elsewhere. These soldiers received influenza vaccinations in basic training. Civilian employees and soldiers' families were offered vaccine, but only an estimated <40% accepted (*4*).

Training stopped over the Christmas–New Year's holidays and resumed on January 5, 1976, with an influx of new trainees. The weather was cold (wind chill factors of 0° to –43°F), and the reception center was crowded (*4*). Resumption of training was associated with an explosive febrile respiratory disease outbreak involving new arrivals and others. Throat swabs were collected from a sample of hospitalized soldiers with this syndrome. On January 23, the Fort Dix preventive medicine physician learned of 2 isolations of adenovirus type 21 and suspected an adenovirus outbreak (*4*). He notified the county health department and the New Jersey (NJ) Department of Health of the outbreak (*4*). On January 28, an NJ Department of Health official consulted with the military physician and suggested that the explosive, widespread outbreak could be influenza (*4*). Over the next 2 days, 19 specimens were delivered to the state laboratory and 7 A/Victoria-like viruses and 3 unknown hemagglutinating agents were identified (*4*). Specimens were flown to the Center for Disease Control (CDC), Atlanta, Georgia, on February 6, where a fourth unknown agent was found (*4*).

On February 2, Fort Dix and NJ Department of Health personnel arranged for virologic studies of deaths possibly caused by influenza (*4*). Tracheal swabs taken on February 5 from a recruit who died on February 4 yielded a fifth unknown agent on February 9. By February 10, laboratory evidence had confirmed that a novel influenza strain was circulating at Fort Dix and that 2 different influenza strains were causing disease. By February 13, all 5 unknown strains were identified as swine influenza A (Hsw1N1). The possibility of laboratory contamination was evaluated (*4*). No known swine influenza A strains were present in the NJ Department of Health Virus Laboratory before the Fort Dix outbreak. Additionally, all unknown Fort Dix viruses were independently isolated from original specimens at CDC and the Walter Reed Army Institute of Research (WRAIR), Washington, DC. Also, 2 patients with novel virus isolates had convalescent-phase, homologous, hemagglutination-inhibition (HAI) antibody titers of 1:40–1:80, consistent with recent infections. The new influenza strain had been independently identified in 3 different laboratories and supporting serologic evidence developed within 15 days after the original specimens were collected (Table) (*4*).

**Table:** Key events in the swine influenza A (Hsw1N1) outbreak, Fort Dix, NJ

Date (1976)	Event
January 5	After the holidays, basic training resumed at Fort Dix, NJ; a sudden, dramatic outbreak of acute respiratory disease followed the influx of new recruit trainees (*4*).
January 19	Earliest hospitalization of a Fort Dix soldier with acute respiratory disease attributed to swine influenza A (Hsw1N1) (identified retrospectively by serologic tests) (*7**,**14*)
January 21	Influenza A/Victoria (H3N2) identified away from Fort Dix in NJ civilians (*4*)
January 23	Fort Dix received reports of adenovirus type 21 isolations from soldiers ill with respiratory disease and reported the outbreak to the local and state health departments (*4*)
January 28	A NJ Department of Health official suggested the Fort Dix outbreak may be due to influenza and offered to process specimens for virus isolation (*4*)
January 29–30	19 specimens sent to NJ Department of Health in 2 shipments (*4*)
February 2–3	NJ Department of Health identified 4 isolates of H3N2-like viruses and 2 unknown hemagglutinating agents in 8 specimens sent on January 29. Fort Dix and NJ Department of Health arranged for the study of deaths possibly due to influenza. NJ Department of Health identified 3 H3N2-like viruses and a third unknown hemagglutinating agent in 11 specimens sent on January 30 (*4*).
February 4	Fort Dix soldier died with acute respiratory disease (*4*).
February 5	Tracheal specimens from the soldier who died on February 4 were sent to NJ Department of Health (*4*).
February 6	NJ Department of Health sent the Fort Dix specimens to Center for Disease Control (CDC), Atlanta, GA; CDC identified a fourth unknown hemagglutinating agent in the Fort Dix specimens (*4*).
February 9	Specimens from the soldier who died on February 4 yielded a fifth unknown hemagglutinating agent (*4*). Last hospitalization of an identified Fort Dix soldier with febrile, acute respiratory disease attributed to swine influenza A (Hsw1N1) (identified retrospectively by serologic tests) (*7**,**14*).
February 10	Laboratory evidence supported 2 influenza type A strains circulating on Fort Dix; 1 was a radically new strain. Prospective surveillance for cases in the areas around Fort Dix was initiated; only cases of H3N2 were found (*4*).
February 13	Review of laboratory data and information found that all 5 unknown agents were swine influenza A strains (later named A/New Jersey [Hsw1N1]); 3 laboratories independently identified the swine virus from original specimens (serologic data supporting swine influenza A virus infection was later obtained from 2 survivors with A/New Jersey isolates) (*4*).
February 14–16	Initial planning meeting between CDC, NJ Department of Health, Fort Dix, and the Walter Reed Army Institute of Research personnel was held in Atlanta, GA. Prospective case finding was initiated at Fort Dix; H3N2 was isolated; Hsw1N1 was not isolated (*7*). Retrospective case finding was initiated by serologic study of stored serum specimens from Fort Dix soldiers who had been hospitalized for acute respiratory disease; 8 new cases of disease due to Hsw1N1 were identified with hospitalization dates between January 19 and February 9 (*7**,**14*).
February 22–24	Prospective case finding was again conducted at Fort Dix; H3N2 virus was isolated but not Hsw1N1 (*7*).
February 27	Thirty-nine new recruits entering Fort Dix February 21–27 gave blood samples after arrival and 5 weeks later; serologic studies were consistent with influenza immunization but not spread of H3N2 virus. None had a titer rise to Hsw1N1 (*11*).
March 19	Prospective surveillance identified the last case of influenza in the areas around Fort Dix; only H3N2 viruses were identified outside of Fort Dix (*4*).

## Swine Influenza A Viruses

The swine influenza A (Hsw1N1) viruses from Fort Dix soldiers were studied at CDC (*5**,**6*). The novel virus was named A/New Jersey/76 (Hsw1N1). Initially, HAI serologic studies of Fort Dix populations were performed at WRAIR by using inactivated A/Mayo Clinic/103/74 (Hsw1N1) antigen from CDC (*7*). The A/Mayo Clinic virus was recovered in 1974 from lung tissue obtained at autopsy from a man with Hodgkin disease who lived on a swine farm (*8*). Later, CDC provided WRAIR with A/New Jersey/76 (Hsw1N1) antigen (*7*).

## Outbreak Investigation Planning

Outbreak investigation plans were developed quickly, and lines of communication and responsibilities were defined. Since a retrospective investigation required extensive serologic studies, a serology laboratory was established at WRAIR and operated 7 days a week. The HAI antibody test, which measured antibody to the hemagglutinin glycoprotein, was used to identify infections (*9*). Variables other than 1976 swine virus infection that might influence HAI titers were identified. Influenza A (H1N1) viruses circulated from 1918 to 1957 (*3*). Additionally, earlier military influenza vaccines (1955–1969) and some civilian formulations (1956–1958) contained swine antigens (*10*). Most basic training soldiers were in their late teens and early twenties, so few had potential exposure to military vaccines (the earlier military vaccines were available to civilian workers and soldiers' families) (*10*). Other populations were expected to have age-related antibody from infections or vaccines. Development of heterotypic antibody after vaccination or infection with contemporary H3N2 antigens was possible; populations suitable for assessing this were studied. None of the potential HAI test limitations was considered serious.

The NJ Department of Health continued to provide virus isolation services to the military (*4*). Army personnel investigated the outbreak on Fort Dix; civilian health departments defined the outbreak beyond Fort Dix. CDC provided reference laboratory support and consultation.

## Case Finding at Fort Dix

Case-finding was conducted prospectively and retrospectively (Table). Prospectively, throat washings were collected from patients with febrile, acute respiratory disease who were hospitalized or sought treatment at the emergency room February 14–16 (phase I, n = 50) and February 22–24 (phase II, n = 45) (*7*). Attempts were made to obtain paired serum specimens from phase I patients. Specimens were obtained from 60 basic training soldiers, 13 other military personnel, and 22 civilians. A/Victoria/75 (H3N2) virus was isolated from 34 (68%) persons during phase I and 21 (47%) in phase II (*7*). A/New Jersey/76 (Hsw1N1) was not isolated from any of the 95 patients. One of 34 (3%) persons with an A/Victoria isolate and paired serum samples had a >4-fold rise in titer to A/Mayo Clinic (Hsw1N1) antigen, with an acute titer of <1:10 increasing to 1:20 (*7*).

Retrospective study was made possible by an ongoing Adenovirus Surveillance Program, which collected weekly throats swabs and paired serum specimens from a sample (≈3%–6%) of basic trainees hospitalized with respiratory disease (*7*). Specimens had been sent to Army regional laboratories, and 80% of the paired serum specimens from Fort Dix trainees hospitalized between November 1, 1975, and February 14, 1976, went to Fort Meade, Maryland. Serum specimens not depleted by routine studies were stored. Stored serum specimens from 74 Fort Dix trainees were identified at Fort Meade and forwarded to WRAIR; 39 (53%) of the trainees had been hospitalized after January 1, 1976. These serum samples were initially tested against A/Mayo Clinic antigen. Serum samples with >4-fold rises in titer were re-tested against A/New Jersey and A/Victoria/3/75 (H3N2) antigens (*7*). HAI titers to A/Mayo Clinic and A/New Jersey differed only slightly.

Concerns that influenza A (H3N2) infection or vaccination might stimulate antibody to A/Mayo Clinic were addressed. Four groups were studied to identify persons with >4-fold heterotypic HAI antibody increases to A/Mayo Clinic. None were found in 39 Fort Dix soldiers who received influenza vaccine in February 1976 (group 1), and none were found among 27 hospitalized soldiers from posts other than Fort Dix who had >4-fold rises in complement fixation (CF) antibody to influenza A (group 2) (*7*). In the third group, >4-fold rises in antibody titers developed in 3 (8%) of 40 soldiers from Fort Dix and elsewhere who had been hospitalized with an A/Victoria isolate (*7*). In the fourth group, a single serum sample was studied from each of 168 randomly selected Fort Dix basic trainees who had received their annual influenza vaccination 3 to 4 weeks earlier (*11*). Only 4 (2%) had HAI titers >1:20 to A/Mayo Clinic (*11*). In similar studies by others, in 0%–6% of persons, heterotypic antibody to influenza A/swine developed after infection with A/Victoria (H3N2) or influenza vaccination (*12**,**13*).

Since heterotypic antibody to A/Mayo Clinic seldom occurred, soldiers who were hospitalized for acute respiratory disease and showed a >4-fold titer rise to influenza A (Hsw1N1) in stored serum specimens from the Adenovirus Surveillance Program were considered to have had A/New Jersey infections. Eight new cases in basic trainees were found. Three (38%) of the 8 solders also had >4-fold antibody rises to A/Victoria. Therefore, 13 male, enlisted soldiers, aged 17–21 years, were identified as having had respiratory diseases resulting in hospitalization or death and an A/New Jersey (Hsw1N1) isolate or serologic conversion to A/New Jersey (case-patients). Ten had arrived at Fort Dix between January 5 and February 3, 1976. Three arrived between September 9 and December 30, 1975. Dates of onset of illness were known for 12 and were from January 12 to February 8, 1976. Hospital admissions occurred between January 19 and February 9. Autopsy findings for the only patient who died showed severe edema, hemorrhage, and mononuclear infiltrates in the lungs, consistent with viral pneumonia. No preexisting disease or bacterial infection was found. Four (33%) of the 12 surviving patients had radiologic evidence of pneumonia but their clinical syndromes were similar to those described for patients with infections caused by other influenza A strains (*7*).

Twelve of the 13 patients were basic trainees; one was an office worker who had an A/New Jersey isolate (*7*). The 12 trainees were in 9 different training companies (*7**,**14*). One company had 3 patients, and 1 company had 2 patients. In these 2 companies, all patients came from the same platoon. Nine were interviewed. Except for those in the same unit, the patients were unknown to each other. All denied swine contact for 6 months before admission. No common variables in working or living environments were identified. All had contact with the Fort Dix medical care system, but care took place in 5 clinics and 2 wards. From January 19 to February 9, there were 7 days when none occupied a hospital bed (*7**,**14*).

## Transmission and Illness in Units with Case-patients

Transmission was assessed by using HAI antibody titers to A/Mayo Clinic (Hsw1N1). Sixteen of 17 contacts of the patient not in basic training, 18–43 years of age, were studied, and 4 (25%) had titers >1:20 (*14*). One of the 9 training companies had a case-patient who completed basic training before the case was identified and was not studied. In another company with a case-patient, 13 soldiers were studied, and all had titers <1:10, but their platoons were not identified. Seven companies were studied by comparing the platoon with at least 1 case-patient to other platoons in the company. Some members of all 7 platoons with case-patients had titers >1:20, varying from 7% to 56% (median = 26%). In other platoons from these seven companies, the prevalence of titers >1:20 ranged from 0% to 40% (median 18%), which indicated that A/New Jersey virus transmission was not limited to 1 platoon in most companies (*14*).

Comparable samples of soldiers from the 7 companies with cases discussed above and 7 contemporary companies without cases were evaluated. Prevalences of HAI antibody titers to A/Mayo Clinic >1:20 in the companies with cases ranged from 0% to 45% (median 18%) (*8*). Prevalences in the companies without cases was 0%–10% (median 4%) (*14*).

Available records permitted the identification of hospital admissions for acute respiratory disease in 6 of the 9 companies with an A/New Jersey case. From January 19 to February 9, 1976, when the A/New Jersey patients from these companies were admitted, admission rates for acute respiratory disease of >3.0 per 100 men per week were observed in 4 of the companies. The highest rates occurred during the week ending January 25 and ranged from 1.1 to 6.9 (median 3.4) per 100 men per week (*14*).

## Extent of Spread and Duration of Outbreak

The weekly formation of segregated cohorts of new recruits provided an opportunity to study the extent and duration of virus transmission. A random 9% sample of soldiers beginning basic training from January 5 to March 1 were studied for HAI antibody to A/Mayo Clinic (Hsw1N1) (*11*). The prevalence of titers >1:20 by weekly cohort ranged from 0% to 19%. The 3 highest prevalences, 19%, 12%, and 9%, occurred in cohorts who started training on January 12, 19, and 26, respectively. Prevalences for 6 other cohorts ranged from 0% to 5%, with 0% prevalence in the cohorts that started training on January 5 and March 1 (*11*). Eleven of the 12 Fort Dix basic training soldiers identified as A/New Jersey case-patients also began training on January 12, 19, and 26 (*11**,**14*).

From February 21 to February 27, a total of 39 soldiers in the basic training reception center were studied for HAI antibody to A/New Jersey (Table) (*11*). This same group was studied 5 weeks later. All 39 had HAI antibody titers to A/Mayo Clinic <1:10 initially and at 5 weeks. The prevalence of HAI antibody titers to A/Mayo Clinic antigen was also determined in advanced training students, civilians who visited the Fort Dix Phlebotomy Clinic, installation maintenance workers, basic training instructors, military medical and veterinary personnel, and soldiers who worked in the reception center. In advanced training students and persons <25 years of age, the prevalence of titers >1:20 was 0%–6%, consistent with heterotypic responses. However, titers were higher in persons >26 years old; most had prevalences in the range of 17% to 44%, but women and men >51 years of age at the Phlebotomy Clinic had prevalences of 92% (n = 37) and 88% (n = 60), respectively (*11*).

The earliest A/New Jersey patient was hospitalized on January 19; the last identified patient was admitted on February 9 (Table) (*7*). Both were identified by serologic testing. Four of 5 patients with virus isolates were admitted on January 29 and 30. The last A/New Jersey isolate came from the soldier who died on February 4. The patient admitted on January 19 reported that his onset of illness occurred on January 12. Since no evidence was found for A/New Jersey virus at Fort Dix before January 12, the virus was likely introduced on or shortly after resumption of training on January 5. As shown by the clustering of hospital admissions, the A/New Jersey outbreak peaked during late January and tapered off in early February. The absence of any indication of the A/New Jersey virus in the cohort beginning basic training on March 1 and in the reception center group who gave blood samples from February 21 to February 27 and 5 weeks later supports the conclusion that A/New Jersey disappeared in February (Table) (*11*).

To understand the relationship of the A/Victoria and A/New Jersey/76 (Hsw1N1) outbreaks, serum specimens from the 9% sample of soldiers who began basic training from January 5 to March 1 were also studied for HAI antibody to A/Victoria. The geometric mean titers to A/Victoria >1:10 for cohorts beginning training on January 5 and January 12 were 1:56 and 1:53, respectively. The geometric mean titers then increased to 1:114 in the cohort that started on February 2, peaked at 1:120 in the cohort that began on February 9, remained high at 1:109 for the February 16 cohort, and then returned to baseline (*11*). Thus, the A/New Jersey outbreak likely started in early January and peaked in late January, followed closely by the A/Victoria outbreak.

Even though A/Mayo Clinic titers >1:20 were seen in Fort Dix populations other than basic trainees, the prevalences in young people were very low, consistent with heterotypic antibody. Higher prevalences in older persons could have been related to earlier influenza A (H1N1) infections or vaccinations with vaccines that contained swine influenza antigens (*10*). The high titers to A/Mayo Clinic in these groups could not be related to illness, vaccination, or swine contact (*11*). When the serologic data were extrapolated, the total number of A/New Jersey infections in Fort Dix basic trainees was ≈230 when contacts of all 13 case-patients were considered and ≈142 when only virologically confirmed cases were considered true cases (*11**,**15*).

## Case Finding beyond Fort Dix

Influenza A/Victoria-like strains had been identified in New Jersey as early as January 21, 1976. By the end of January, the state had investigated reports of high employee and student absenteeism and a hospital outbreak. Patients in all episodes were sampled by using virus isolation and serologic testing. All laboratory reports indicated A/Victoria virus infections (*4*).

Starting February 10, arrangements were made to study febrile respiratory disease patients at McGuire Air Force Base (adjoining Fort Dix) and at hospitals, emergency rooms, and physicians' offices in the Fort Dix vicinity. Medical examiners were told to obtain specimens from possible influenza patients and surveillance was increased statewide. From January 9 to March 19, infection with influenza A/Victoria virus was documented in 301 persons by virus isolation (151 persons), CF or HAI serology (113 persons), or both (37 persons). Cases in New Jersey came from 19 of 21 counties, McGuire Air Force Base, and Lakehurst Naval Training Center. Delaware had 19 cases, including 5 from Dover Air Force Base. From January 31 to March 17, 10 civilian deaths in New Jersey were attributed to influenza. Influenza A/Victoria (H3N2) was isolated from all 10 patients (*4*).

The numbers of isolation and serologic specimens tested and the percentages positive for A/Victoria were consistent with an outbreak that began quickly in January and declined in late February to early March. No influenza cases were identified after March 19; influenza A/New Jersey was never isolated outside Fort Dix (Table) (*4**,**7*).

Among patients with serologic evidence of influenza, HAI antibody responses to both A/Victoria and A/New Jersey were studied in 134. Six (4%), aged 22 to 71 years, had >4-fold HAI rises in titer to both viruses (*4*). In the absence of any association with swine influenza A virus, the A/New Jersey titers were attributed to A/Victoria infections.

## Summary and Speculation

A/New Jersey/76 (Hsw1N1) was likely introduced into Fort Dix early in 1976, after the holidays (*15*). The virus caused disease with radiologic evidence of pneumonia in at least 4 soldiers and 1 death; all of these patients had previously been healthy (*7**,**15*). The virus was transmitted to close contacts in the unique basic training environment, with limited transmission outside the basic training group. A/New Jersey probably circulated for a month and disappeared. The source of the virus, the exact time of its introduction into Fort Dix, and factors limiting its spread and duration are unknown (*15*).

The Fort Dix outbreak may have been a zoonotic anomaly caused by introduction of an animal virus into a stressed population in close contact in crowded facilities during a cold winter. However, the impact of A/New Jersey virus on this healthy young population was severe in terms of estimated infections, hospitalizations, and duration of the outbreak.

If the outbreak was more than an anomaly, why did it not extend beyond basic trainees? Several factors merit consideration. Contact between basic trainees and others was limited. Moreover, a swine influenza antigen was included in annual military influenza vaccine formulations from 1955 through 1969 (*10*). The high antibody titers to A/Mayo Clinic antigen observed with increasing age in the Phlebotomy Clinic population may reflect earlier influenza A (H1N1) infections or vaccine exposure and some protection (*11*). Also, competition between A/New Jersey and A/Victoria viruses must be considered. The A/Victoria virus spread widely and may have limited the impact of A/New Jersey virus with its lesser ability for human transmission.

Could the Fort Dix outbreak have resulted from interaction between swine influenza A and A/Victoria viruses? A/Victoria transmission occurred in New Jersey before A/New Jersey was identified at Fort Dix. Is it possible that A/Victoria virus and an early A/New Jersey virus coinfected a soldier with genetic exchange, resulting in a recombinant virus with enhanced human transmission capability? The rapid disappearance of A/New Jersey prohibited studies of virus interactions. Genetic analyses of A/New Jersey, A/Victoria and contemporary A/swine viruses might elucidate a relationship.

Communication and collaboration existed at the onset of the outbreak and continued throughout the investigation. The points of contact at the NJ Department of Health, Fort Dix, CDC, and WRAIR had been established before the outbreak, so time was not lost identifying organizations and persons who needed to be contacted. Organizational roles were defined early and respected. The development of outbreak investigation plans, collaboration in field and laboratory work, and exchange of information occurred smoothly. An important part of the Army investigation was establishment of points of contact at WRAIR who communicated with military leaders, the NJ Department of Health, CDC, and the press. Military epidemiology and laboratory teams reported to WRAIR points of contact. This system protected these teams from disruptive inquiries.

The burden on the laboratories supporting this investigation was intense, lasting for weeks. In 1976, WRAIR was a research and field epidemiology laboratory that also operated as a public health reference laboratory. The WRAIR commander had the authority to reallocate and mobilize scientists and laboratory resources. Today, WRAIR no longer functions as a public health laboratory. The depth of resources and flexibility that existed at WRAIR in 1976 cannot be found in other military laboratories (*16*). Duplicating the 1976 laboratory effort today, in timely fashion, would be difficult.
